# Multi-Loop Model of Alzheimer Disease: An Integrated Perspective on the Wnt/GSK3β, α-Synuclein, and Type 3 Diabetes Hypotheses

**DOI:** 10.3389/fnagi.2019.00184

**Published:** 2019-07-31

**Authors:** Nicholas G. Norwitz, Adrian Soto Mota, Sam G. Norwitz, Kieran Clarke

**Affiliations:** ^1^Department of Physiology, Anatomy and Genetics, University of Oxford, Oxford, United Kingdom; ^2^Department of Neuroscience, Washington University in St. Louis, St. Louis, MO, United States

**Keywords:** Alzheimer disease, Aβ, α-synuclein, GSK3β, Parkinson’s disease, tau, type 3 diabetes, Wnt-signaling

## Abstract

As the prevalence of Alzheimer disease (AD) continues to rise unabated, new models have been put forth to improve our understanding of this devastating condition. Although individual models may have their merits, integrated models may prove more valuable. Indeed, the reliable failures of monotherapies for AD, and the ensuing surrender of major drug companies, suggests that an integrated perspective may be necessary if we are to invent multifaceted treatments that could ultimately prove more successful. In this review article, we discuss the Wnt/Glycogen Synthase Kinase 3β (GSK3β), α-synuclein, and type 3 diabetes hypotheses of AD, and their deep interconnection, in order to foster the integrative thinking that may be required to reach a solution for the coming neurological epidemic.

## Introduction

Alzheimer disease (AD) is among the most ominous of modern health epidemics. The current costs, both human and financial, are staggering and climbing at a precipitous rate. In the United States alone, 5.5 million adults live with AD, imposing an economic burden of $259 billion (Alzheimer’s Association, 2017). Over the next three decades, the number of people living with AD is expected to triple to 13.8 million and the economic costs are projected to quadruple to $1.1 trillion, single-handedly crippling the United States health care system. AD is also the only disease on the list of the top 10 disease causes of death for which there is currently no effective treatment (Alzheimer’s Association, 2017).

AD is not alone in its ascent. Other chronic diseases, particularly Parkinson’s disease (PD), a neurodegenerative disorder associated with the build-up of α-synuclein protein and death of dopaminergic neurons, and type 2 diabetes mellitus (T2DM) are increasing in prevalence at similarly alarming rates (Boyle et al., 2010; Rocca, 2018). Although AD, PD, and T2DM share common risk factors, chief among these being age, there is more to their relationship. Evidence suggests that the pathophysiological mechanisms underlying AD, PD, and T2DM interact synergistically (Giasson et al., 2003; de la Monte and Wands, 2008; Duka et al., 2009; Wills et al., 2010; Gao et al., 2012; Gąssowska et al., 2014; Roberts et al., 2017; Yan et al., 2018).

In addition to the well-known amyloid cascade hypothesis of AD, other hypotheses have been proposed that include: (1) the Wnt/Glycogen Synthase Kinase 3β (GSK3β) hypothesis (Hooper et al., 2008; De Ferrari et al., 2014; Llorens-Martín et al., 2014), (2) the α-synuclein hypothesis (Moussaud et al., 2014; Yan et al., 2018), and (3) the type 3 diabetes hypothesis (de la Monte and Wands, 2008). In this review article, we focus on the Wnt/GSK3β hypothesis, describing how it serves as a platform for a set of positive feedback loops that contribute to the pathogenesis of AD. In turn, we also discuss the α-synuclein and type 3 diabetes hypotheses, describing how they each constitute their own feedback loops and interact with the Wnt/GSK3β model.

## Wnt/GSK3β

### Overview of Wnt-Signaling

**Figure 1:**
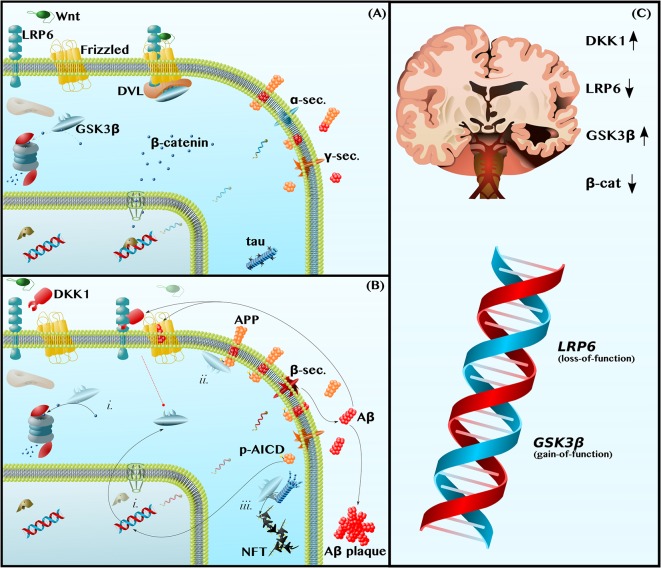
Dysfunctions in canonical Wnt-Signaling contribute to the neuropathology of Alzheimer disease (AD). **(A)** Functional Wnt and Nonamyloidogenic Processing—Glycogen Synthase Kinase 3β (GSK3β) is a constitutively active kinase that phosphorylates and targets β-catenin for proteasomal degradation. The binding of Wnt ligands to LDL Receptor-Related Protein 6 (LRP6) and Frizzled induces the receptor pair to bind Dishevelled (DVL), a protein that serves as a docking platform for GSK3β. The sequestration of GSK3β by the Wnt receptor complex permits β-catenin to accumulate and translocate into the nucleus, where it binds transcription factors to induce gene expression. This includes promoting anti-amyloidogenic α-secretase expression (blue mRNA) and inhibiting pro-amyloidogenic β-secretase expression. **(B)** Dysfunctional Wnt, Amyloidogenic Processing, and Tau Hyperphosphorylation—The LRP6 antagonist, Dickkopf 1 (DKK1), prevents Wnt-induced GSK3β inhibition (broken red line). Thus, (i) GSK3β causes β-catenin depletion, contributing to a decrease in α-secretase expression and increase in β-secretase expression (red mRNA). In addition, (ii) GSK3β phosphorylates the intracellular domain of Amyloid Precursor Protein (APP), making APP a better substrate for β-secretase and further promoting amyloidogenic processing and the production of Amyloid β (Aβ) by β- and γ-secretase. Aβ inhibits Frizzled and induces DKK1 expression to feedback and prevent GSK3β inhibition (broken red line). Aβ also forms extracellular plaques. The leftover phosphorylated APP Intracellular Domain (p-AICD) induces GSK3β expression. Finally, (iii) GSK3β, also known as Tau Kinase I, phosphorylates tau, contributing to microtubule instability and to the formation of neurotoxic oligomers and phospho-tau (p-tau) Neurofibrillary Tangles (NFTs). **(C)** Human Neuropathological and Genetic Data are Consistent with the Wnt/GSK3β Model of AD—In the Alzheimer brain, as compared to the healthy aged brain, the levels and activities of Wnt-signaling components are indicative of pathway hypoactivity: DKK1 levels are elevated, LRP6 levels are reduced, GSK3β activity is high, and β-catenin is depleted. Furthermore, *LRP6* loss-of-function and *GSK3β* gain-of-function alleles are risk factors for AD.

Wnt-signaling refers to a set of highly conserved signal transduction pathways that are widely expressed throughout the body and that play a vital role both in neuronal development and in the maintenance of proper neuronal function in the adult human brain (Patapoutian and Reichardt, 2000; Oliva et al., 2013; Rosso and Inestrosa, 2013; Nusse and Clevers, 2017). In this article, we focus on the better-studied canonical Wnt-β-catenin-signaling pathway, leaving the topic of the two non-canonical Wnt-signaling pathways (the Wnt-planar cell polarity and Wnt-calcium pathways) for others to discuss in depth (Mudher et al., 2001; Gao et al., 2012; Oliva et al., 2013; Rosso and Inestrosa, 2013; Wan et al., 2014). Canonical Wnt-β-catenin-signaling (hereafter, referred to simply as Wnt-signaling) is initiated by the binding of Wnt ligands to the Wnt receptor pair, Low-Density Lipoprotein Receptor-Related Protein 6-Frizzled (LRP6-Fz). LRP6 then recruits Dishevelled (DVL), a scaffolding protein that sequesters GSK3β from the cytoplasm. The inhibition of GSK3β, a constitutively active kinase that targets the transcriptional cofactor β-catenin for proteasomal degradation, is central to Wnt-signaling. Simply put, Wnt-signaling inhibits GSK3β, permitting β-catenin to accumulate in the cytoplasm and translocate into the nucleus to mediate the transcription of genes, such as *BACE1* and *ADAM10* (elaborated upon below), involved in the pathogenesis of AD (Rosso and Inestrosa, 2013; Figure 1A).

### Dysfunctional Wnt-Signaling Causes the Production of Aβ

Amyloid plaques, aggregates of the amyloid β (Aβ) peptide, are the primary pathological hallmark of AD. Aβ is formed by the sequential cleavage of amyloid precursor protein (APP) by β- and γ-secretase (Figure 1B). This amyloidogenic processing is in contrast to nonamyloidogenic processing, in which α-secretase replaces β-secretase and cleaves APP within the Aβ domain such that no Aβ is produced (Haass et al., 2012; Figure 1A). By altering α- and β-secretase gene expression, as well as by decreasing APP phosphorylation, Wnt-signaling shifts APP metabolism away from amyloidogenic processing and protects against Aβ neuropathology (Alvarez et al., 2004; Parr et al., 2012; Liu et al., 2014; De Ferrari et al., 2014; Llorens-Martín et al., 2014; Wan et al., 2014).

With respect to secretase gene expression, Wnt-signaling downregulates the sole β-secretase gene, *BACE1* (Haass et al., 2012; Parr et al., 2015), and upregulates the primary neuronal α-secretase gene, *ADAM10* (Haass et al., 2012; Wan et al., 2012). In APP-overexpressing mice, GSK3β inhibition has been shown to decrease *BACE1* expression and activity, thereby reducing amyloid plaque load (Ly et al., 2013). Furthermore, in cultured neurons, activating Wnt-signaling, by using Wnt ligands or overexpressing β-catenin, is sufficient to increase *ADAM10* expression (Wan et al., 2012) and decrease *BACE1* expression, again reducing Aβ levels (Parr et al., 2015). These data are consistent with a model in which dysfunctional Wnt-signaling in the AD brain causes GSK3β-mediated β-catenin depletion, which leads to a pathological decrease in the ratio of α-secretase to β-secretase expression (Figure 1Bi) and, thus, to an increase in the amyloidogenic processing of APP to Aβ (Mudher et al., 2001; Chami et al., 2012; Wan et al., 2012; Ly et al., 2013; De Ferrari et al., 2014; Llorens-Martín et al., 2014; Wan et al., 2014; Golpich et al., 2015; Parr et al., 2015).

Wnt-signaling further suppresses amyloidogenic processing by inhibiting APP phosphorylation. Specifically, Wnt-signaling inhibits GSK3β, which otherwise phosphorylates APP on Thr668 (Saeki et al., 2011; Acevedo et al., 2014), contributing to the elevated p-Thr668 APP levels that are observed in the human AD brain (Lee et al., 2003; Figure 1Bii). The direct consequences of Thr668 phosphorylation are two-fold. First, p-Thr668 APP is a better substrate for β-secretase than unphosphorylated APP (Lee et al., 2003). Second, if the APP intracellular domain (AICD)—which contains Thr668 and is produced in conjunction with Aβ by γ-secretase-mediated cleavage—is phosphorylated, it can translocate into the nucleus to upregulate *GSK3β* gene expression (Chang et al., 2006; Figure 1B). In this way, dysfunctional Wnt-signaling permits the phosphorylation of APP by GSK3β, leading to both an increase in Aβ production and an increase in *GSK3β* expression, establishing a positive feedback loop.

As predicted by this model, inhibiting Wnt-signaling with the LRP6 inhibitor, Dicckopf-1 (DKK1), increases the amyloidogenic processing of APP and impairs learning and memory in mice (Killick et al., 2012; Parr et al., 2015; Marzo et al., 2016; Elliott et al., 2018; Sellers et al., 2018), whereas activating Wnt-signaling with different GSK3β inhibitors decreases *BACE1* expression, decreases APP phosphorylation, decreases Aβ production, prevents neurodegeneration, and reduces learning and memory (Ryder et al., 2003; Chang et al., 2006; Rockenstein et al., 2007; Fiorentini et al., 2010; Toledo and Inestrosa, 2010; Ly et al., 2013; Pan et al., 2018).

### Aβ Causes Dysfunctional Wnt-Signaling

Aβ, in turn, can inhibit Wnt-signaling to establish another positive feedback loop. Treatment of rat neurons *in vitro* with Aβ induces the expression of DKK1 and increases GSK3β activity, thereby decreasing β-catenin levels and contributing to the death of neurons (Alvarez et al., 2004; Caricasole et al., 2004; Killick et al., 2012; Elliott et al., 2018; Sellers et al., 2018). Importantly, activation of Wnt-signaling via a variety of mechanisms—by treatment with Wnt ligands, neutralization of DKK1, or inhibition of GSK3β—appears sufficient to protect neurons against β-catenin depletion and, ultimately, death (Alvarez et al., 2004; Caricasole et al., 2004; Silva-Alvarez et al., 2013).

Not only does Aβ indirectly inhibit the initiation of Wnt-signaling by increasing the expression of the LRP6 antagonist, DKK1 (Caricasole et al., 2004; Killick et al., 2012; Purro et al., 2012; Marzo et al., 2016; Elliott et al., 2018), but it also directly blocks the binding of Wnt ligands to the other half of the LRP6-Fz receptor pair. Using cultured mouse neurons, Magdesian et al. (2008) demonstrated that Aβ competitively inhibits the binding of Wnt ligands to Fz and, consequently, prevents β-catenin from translocating into the nucleus to induce Wnt target gene expression (Figure 1B). Aβ also increases GSK3β activity leading to neurodegeneration (Alvarez et al., 2004; Caricasole et al., 2004; Hooper et al., 2008; De Ferrari et al., 2014; Llorens-Martín et al., 2014; Wan et al., 2014). Importantly, interventions that either block the interaction between Aβ and the Wnt receptors, or those that circumvent the Aβ blockade and activate Wnt-signaling downstream of LRP6-Fz, protect neurons against Aβ toxicity (Alvarez et al., 2004; Caricasole et al., 2004; Hooper et al., 2008; Magdesian et al., 2008; De Ferrari et al., 2014; Llorens-Martín et al., 2014; Wan et al., 2014). Some examples are as follows: a synthetic soluble peptide homologous to Fz competitively inhibited Aβ binding to Fz and, thereby, protected against β-catenin depletion (Magdesian et al., 2008); upstream activation of Wnt-signaling using competitive amounts of exogenous Wnt ligands (Wnt3a or Wnt7a) prevented Aβ-induced neuron apoptosis; downstream activation of Wnt-signaling using multiple different GSK3β inhibitors also prevented Aβ-induced neurodegeneration (Alvarez et al., 1999, 2004; Silva-Alvarez et al., 2013).

### An LRP6 Deletion Model Supports the Wnt/GSK3β-Aβ Feedback Loop

An *LRP6* deletion mouse model provides further support for the hypothesis that dysfunctional Wnt-signaling and Aβ constitute two halves of a positive feedback loop. Liu and coworkers demonstrated that conditional deletion of *LRP6* in mouse neurons increased levels of β-secretase cleavage products and precipitated the formation of Aβ plaques, consistent with the notion that decreased Wnt-signaling promotes the formation of amyloid pathology. The neuropathological changes were associated with significant memory deficits, similar to those exhibited by more common mouse models of AD (Liu et al., 2014). Importantly, Aβ, in turn, decreased *LRP6* expression, thus validating the positive feedback loop model in which dysfunctional Wnt-signaling causes an increase in Aβ, and vice versa.

These mouse data paralleled those from human patients with AD. Liu et al. not only found (1) lower LRP6 and β-catenin levels in the post-mortem brains of AD patients relative to age-matched control brains (Figure 1C), but also (2) a negative correlation between LRP6 and Aβ levels in these brains and (3) a positive correlation between LRP6 levels and Mini-Mental State Examination (MMSE) scores, a test in which higher scores indicate better cognitive function (Liu et al., 2014). Thus, the level of Wnt-signaling dysfunction may predict the degree of neuropathology and cognitive impairment in AD patients.

### Human Neuropathological and Genetic Data Support the Wnt/GSK3β Model

Not only are LRP6 levels reduced in the post-mortem brains of AD patients, but DKK1 levels are also elevated (Caricasole et al., 2004; Oliva et al., 2013; Wan et al., 2014). The simultaneous decrease in the Wnt receptor (LRP6) and increase in its inhibitor (DKK1) cooperatively downregulates Wnt-signaling and increases GSK3β activity in patients’ brains (Leroy et al., 2007; Hooper et al., 2008; Oliva et al., 2013; Llorens-Martín et al., 2014; Wan et al., 2014; Lazzara and Kim, 2015). The genetic data concur. Specifically, a loss-of-function mutation in *LRP6* has been identified as a risk factor for AD (De Ferrari et al., 2007), as have gain-of-function mutations in the *GSK3β* gene (Schaffer et al., 2008; Figure 1C).

Furthermore, evidence suggests that the strongest known genetic risk factor for AD in humans, the *ApoE4* allele (Liu et al., 2013), may negatively impact Wnt-signaling. Similar to Aβ, the ApoE4 protein increases DKK1 expression, binds to the LRP6-Fz receptor complex, activates GSK3β, and promotes the amyloidogenic processing of APP (Kim et al., 1998; Cedazo-Mínguez et al., 2003; Caruso et al., 2006; Chami et al., 2012; De Ferrari et al., 2014; Wan et al., 2014; Theendakara et al., 2016). Therefore, there is a case to be made that ApoE4 either sparks the positive feedback loop between Wnt-signaling and Aβ, decreases the threshold for the establishment of the feedback loop, and/or accelerates the rate at which the loop spirals into life-altering disease.

### GSK3β Links Aβ to p-tau

In addition to contributing to the build-up of amyloid plaques, the first of the two pathological hallmarks of AD, dysfunctional Wnt-signaling may also contribute to the development of the second hallmark of AD, phospho-tau (p-tau) Neurofibrillary Tangles (NFTs). GSK3β, alternatively known as Tau Kinase I, is thought to be the mechanistic link between Aβ and p-tau (Lucas et al., 2001; Leroy et al., 2007; Saeki et al., 2011; De Ferrari et al., 2014; Llorens-Martín et al., 2014). By inhibiting Wnt-signaling, Aβ increases GSK3β activity (Alvarez et al., 2004; Caricasole et al., 2004; Hooper et al., 2008; De Ferrari et al., 2014; Llorens-Martín et al., 2014; Wan et al., 2014). In turn, GSK3β phosphorylates tau on a set of residues known to be phosphorylated in AD (Lucas et al., 2001; Leroy et al., 2007; Saeki et al., 2011; De Ferrari et al., 2014; Llorens-Martín et al., 2014). This results in two events. First, tau dissociates from microtubules, disabling tau’s physiological function as a microtubule-associated protein and thereby contributing to cytoskeleton instability [as an aside, it’s worth noting that recent data suggest tau functions as more than just a microtubule-associated protein and that tau loss-of-function can contribute to a broader array of cellular defects than previously thought, including brain insulin resistance (Marciniak et al., 2017)]. Second, hyperphosphorylated tau aggregates into neurotoxic oligomers that exert further harmful effects on the cell, such as inducing mitochondrial dysfunction, oxidative stress, neuroinflammation, and apoptosis (Götz et al., 2013; Nilson et al., 2017; Shafiei et al., 2017; Figure 1Biii).

Experiments conducted in two different animal models of AD, GSK3β mice and APP mice, build a strong case for the serial connection amongst Aβ, GSK3β, and p-tau. First, conditional overexpression of *GSK3β* in the cortices and hippocampi of adult mice has been shown to reduce levels of nuclear β-catenin and increase levels of p-tau (Lucas et al., 2001). The GSK3β-induced increase in p-tau pathology is further associated with an increase in neuronal apoptosis and performance deficits in the Morris water maze test of spatial memory (Lucas et al., 2001; Hernández et al., 2002). Second, mice overexpressing APP have increased Aβ and p-tau loads, along with memory deficits. However, inhibition of GSK3β in these APP mice is sufficient to protect against p-tau pathology and against cognitive impairment (Rockenstein et al., 2007). The neuroprotective and anti-p-tau effects of GSK3β inhibition in the APP mouse model have been replicated by multiple independent groups (Fiorentini et al., 2010; Pan et al., 2018). In short, the two murine models suggest that GSK3β/Tau Kinase I, a central player in Wnt-signaling, links the Aβ and p-tau pathologies of AD.

## α-Synuclein

### Human Neuropathological and Genetic Data Suggest Overlapping Pathology Between AD and PD

Neither AD nor PD are monolithic disease entities; it is likely that each is composed of several subtypes that have yet to be effectively characterized. At least some of the putative AD subtypes overlap in pathology with those of PD, and vice versa. More than half of patients with AD present with Lewy bodies, aggregates of α-synuclein that are the PD equivalent of Aβ plaques (Moussaud et al., 2014; Yan et al., 2018). Furthermore, α-synuclein is a component of AD plaques themselves. In fact, the creatively named non-Aβ component (NAC) of plaques is a fragment of α-synuclein (Uéda et al., 1993; Jakes et al., 1994). Thus, α-synuclein lesions are present in the AD brain as distinct Lewy body structures and as part of amyloid plaques.

Complementarily, classic AD inclusions are observed in the PD brain. Specifically, in PD patients, p-tau tends to aggregate in the substantia nigra and other PD-associated brain regions (Kotzbauer et al., 2004; Wills et al., 2010; Moussaud et al., 2014; Yan et al., 2018). This presence of p-tau tangles also correlates with increased GSK3β activity, an observation that suggests GSK3β may be responsible for tau phosphorylation in PD, as it is in AD (Duka et al., 2009; Nagao and Hayashi, 2009; Wills et al., 2010; Golpich et al., 2015; Lazzara and Kim, 2015). An extension of this logic is that dysfunctional Wnt-signaling may be a convergence point for the world’s two most common neurodegenerative disorders.

The genetic evidence also suggests that GSK3β, tau, and α-synuclein can synergistically interact in neurodegeneration. As in AD, polymorphisms in the genes that code for GSK3β and tau (*MAPT*) are risk factors for PD (Kwok et al., 2005; Goris et al., 2007; Schaffer et al., 2008; Moussaud et al., 2014; Golpich et al., 2015). Furthermore, there is a genetic interaction between *MAPT* and the α-synuclein gene (*SNCA*) in which the high-expression *MAPT* haplotype (H1) and a polymorphism in *SNCA* synergistically increase PD risk (Goris et al., 2007). Notably, in this study, only PD patients with the H1/H1 *MAPT* haplotype went on to develop PD with dementia, hinting that this may be an instance in which the pathology and symptoms of a PD subtype overlap with those more typical of AD (Goris et al., 2007).

More relevant to this review article, the *SNCA* gene also affects AD risk. Some *SNCA* polymorphisms double the risk of AD (Matsubara et al., 2001; Wang et al., 2016), whereas others decrease the risk of AD (Xia et al., 1996). With respect to the latter, a retrospective study conducted by Xia et al. (1996) showed that a particular allele in the *SNCA* promoter was enriched 4-fold in cognitively healthy *ApoE4* carriers as compared to *ApoE4* carriers with AD, suggesting that this *SNCA* polymorphism has a protective effect against the strongest known risk factor for AD. This interaction was dose-dependent as the presence of the *SNCA* allele decreased AD risk by 3-fold in *ApoE4* heterozygotes and by 10-fold in *ApoE4* homozygotes (Xia et al., 1996). The fact that *SNCA* mutations affect AD risk is consistent with the hypothesis that α-synuclein plays a role in the development of AD, at least in some instances.

### α-Synuclein Induces Amyloid Pathology, Possibly in a Wnt/GSK3β-Dependent Manner, and Is in Positive Feedback With Aβ

**Figure 2:**
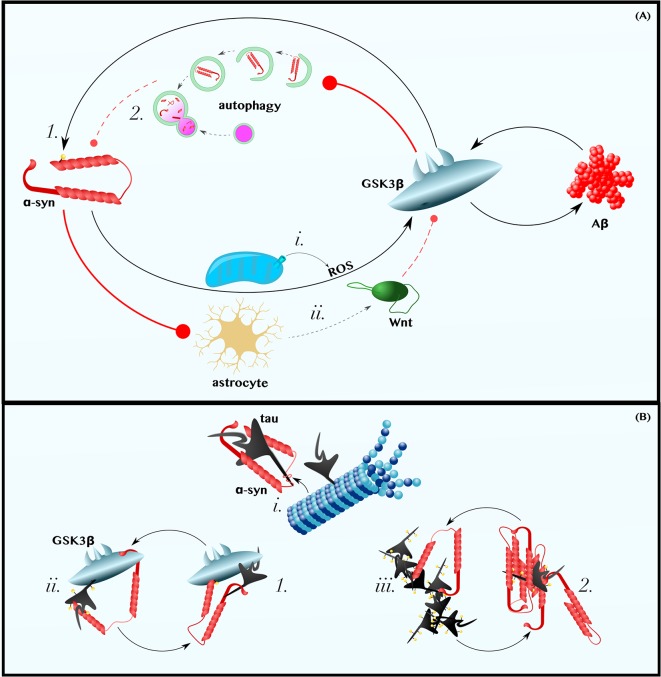
α-Synuclein is in positive feedback with the Aβ and tau pathologies of AD. **(A)** α-Synuclein is in Positive Feedback with Aβ—α-synuclein may induce oxidative stress and promote astrocytic dysfunction. Thus, perhaps by (i) increasing the levels of cytoplasmic ROS and (ii) decreasing those of extracellular astrocyte-derived Wnt ligands, α-synuclein activates GSK3β and induces the production of Aβ (for a more comprehensive discussion about the role of Wnt-signaling in neuron-glia crosstalk in neurodegeneration, see L’Episcopo et al., 2018). In turn, Aβ activates GSK3β, which (1) phosphorylates α-synuclein on Ser129 and (2) may impair the autophagic clearance of α-synuclein. **(B)** α-Synuclein is in Positive Feedback with Tau—α-synuclein can (i) bind tau’s microtubule-binding domain, causing tau to disassociate from microtubules, (ii) recruit GSK3β to tau and, thereby, promote tau hyperphosphorylation, and (iii) directly seed or chaperone the pathological aggregation of p-tau. Tau can reciprocate by (1) recruiting GSK3β to α-synuclein, thereby permitting pathogenic Ser129 phosphorylation, and by (2) promoting the aggregation of α-synuclein. Dashed and solid lines indicate regulatory mechanisms that are, respectively, impaired and enhanced in AD.

Studies using cultured neurons have demonstrated that either exogenous treatment with α-synuclein or α-synuclein overexpression is sufficient to increase the production and secretion of Aβ (Majd et al., 2013; Roberts et al., 2017). One mechanism by which α-synuclein could increase Aβ levels is by activating GSK3β, as suggested by mouse experiments that show that α-synuclein overexpression increases GSK3β activity (Duka et al., 2009; Golpich et al., 2015). Exactly how α-synuclein activates GSK3β is a matter that requires further investigation; however, several lines of *in vitro* and mouse data imply that α-synuclein in neurons could induce GSK3β-activating ROS (Xu et al., 2002; Witt and Flower, 2006; Wakatsuki et al., 2011, 2015; Perfeito et al., 2017; Figure 2Ai) and decrease the production neuroprotective canonical Wnt ligands by astrocytes (L’Episcopo et al., 2011, 2013, 2014; Okamoto et al., 2011; Lindström et al., 2017; Liu et al., 2018; Figure 2Aii; for an excellent review of the role of Wnt-signaling in neuron-microglia-astrocyte crosstalk in neurodegeneration, see L’Episcopo et al., 2018). Although the dominant mechanism by which α-synuclein induces GSK3β *in vivo* is unclear, the observation that intracranial injections of α-synuclein increase β-secretase and Aβ levels in mice (Roberts et al., 2017) is, at minimum, consistent with the model presented in Figure 1B and with the hypothesis that α-synuclein-induced Aβ production is mediated by the Wnt/GSK3β axis.

In turn, exogenous treatment with Aβ, even at concentrations as low as 1 μM, has been shown to increase α-synuclein levels in neurons (Majd et al., 2013). Although the mechanisms by which Aβ reciprocally induces α-synuclein likewise remains a gap in the literature, it is worth noting that upregulation of Wnt-signaling via β-catenin overexpression or GSK3β inhibition protects PD models from developing α-synuclein pathology and motor deficits (Yuan et al., 2015; Stephano et al., 2018). Furthermore, *in vitro*, *Drosophila*, mouse, and human data collectively suggest that GSK3β specifically phosphorylates Ser129 of α-synuclein (Figure 2A1), a post-translational modification predominant in Lewy bodies and in the PD brain that may enhance α-synuclein aggregation and/or neurotoxicity (Fujiwara et al., 2002; Chen and Feany, 2005; Anderson et al., 2006; Credle et al., 2015). GSK3β is also a known inhibitor of autophagy (Parr et al., 2012; Ren et al., 2016; Weikel et al., 2016), a ubiquitous cellular recycling process required for the effective clearance of excess α-synuclein (Vogiatzi et al., 2008; Sato et al., 2018; Figure 2A2). Therefore, it is plausible that Aβ-induced GSK3β activation (Figure 1B) completes an α-synuclein-Aβ feedback loop relevant in some cases of AD.

### α-Synuclein, Directly and via GSK3β, Induces Tauopathy and Is in Positive Feedback with p-tau

α-synuclein and tau interact directly (Jensen et al., 1999; Yan et al., 2018). Specifically, α-synuclein binds tau within tau’s microtubule-binding domain (Jensen et al., 1999). Even were this interaction not sufficient to cause tau to disassociate from microtubules, the binding of α-synuclein to tau induces the phosphorylation of tau on Ser262, a post-translational modification observed in the AD brain that causes tau to release from microtubules, contributing to cytoskeleton instability (Jensen et al., 1999). Subsequently, α-synuclein can serve as a necessary cofactor to help p-tau form oligomers and, eventually, tangles (Giasson et al., 2003; Cremades et al., 2012). Thus, as reviewed by Moussaud et al. (2014), there are at least three ways by which α-synuclein can instigate and aggravate tauopathy: by blocking the interaction between tau and microtubules, thereby interfering with tau’s physiological function (Figure 2Bi), by recruiting kinases that promote tau hyperphosphorylation (Figure 2Bii), and by seeding or chaperoning the aggregation of tau into neurotoxic oligomers andfibrils (Figure 2Biii).

With regard to the kinase mechanism listed above, GSK3β/Tau Kinase I may play a particularly important role in the relationship between α-synuclein and tau. Not only does α-synuclein interact with tau, but both proteins also interact with, and are phosphorylated by, GSK3β (Duka et al., 2009; Credle et al., 2015). Thus, α-synuclein can recruit GSK3β to tau, leading to tau hyperphosphorylation (Figure 2Bii). As this model predicts, exogenous treatment of cultured cells with α-synuclein increased levels of p-tau, this phenomenon being blocked by the inhibition of GSK3β (Gąssowska et al., 2014). Similar findings have been produced in mice in which the overexpression of α-synuclein is sufficient to induce GSK3β-mediated p-tau pathology (Duka et al., 2009). Reflecting on the stimulatory effect of α-synuclein on GSK3β, as well as that of Aβ on GSK3β (Figure 1B), we can elaborate upon our model: GSK3β can be conceptualized as the convergence point of a Y-shaped cascade in which either Aβ or α-synuclein can activate and/or recruit GSK3β to induce tau pathology.

Similar to the mutualistic case of Aβ and α-synuclein, p-tau can promote α-synuclein pathology (Giasson et al., 2003; Badiola et al., 2011; Yan et al., 2018). Using multiple different cell models, Badiola et al. (2011) demonstrated that tau enhanced the aggregation of α-synuclein. In these experiments, tau overexpression also reduced cell viability in an α-synuclein-dependent manner (Badiola et al., 2011), perhaps by promoting the GSK3β-mediated neurotoxic phosphorylation of α-synuclein on Ser129 (Fujiwara et al., 2002; Chen and Feany, 2005; Anderson et al., 2006; Credle et al., 2015), and promoted the secretion of α-synuclein (Badiola et al., 2011). Thus, tau can complete an intracellular positive feedback loop with α-synuclein, possibly by facilitating the pathogenic phosphorylation of α-synuclein Ser129 by GSK3β (Figure 2B1) and/or by promoting α-synuclein’s aggregation (Figure 2B2), and tau might also support the prionic cell-to-cell propagation of α-synuclein (not shown in Figure 2). Independent of the exact mechanisms, the relevance of tau on α-synuclein pathology and its attending symptoms has been demonstrated *in vivo*. In mice, the transgenic expression of tau enhances the formation of α-synuclein inclusions and the corresponding Parkinsonian phenotype (Giasson et al., 2003).

## Type 3 Diabetes

### Overview of Insulin Signaling and Its Role in the Brain

Several lines of evidence suggest that, in the central nervous system, insulin does much more than promote glucose uptake. Insulin is a neuromodulator, affecting the reuptake and production of particular neurotransmitters (Schulingkamp et al., 2000; Plum et al., 2005); insulin regulates food intake and reproduction by acting on the hypothalamus to alter endocrine system function (Plum et al., 2005); and, glucose transport into neurons is largely insulin-independent. Building upon this last key piece of evidence, neuron energy utilization also correlates poorly with the heterogeneous distribution of Insulin Receptor (IRs) throughout the brain, further suggesting that insulin’s primary functions in the brain include more than glucose uptake (Schulingkamp et al., 2000). And, although IRs are also concentrated in the hypothalamus, olfactory bulb, and cerebellum, it’s notable that IRs are particularly densely packed in the hippocampus and cerebral cortex, two brain regions important in learning and memory that are critically impacted by AD (Marks et al., 1990; Schulingkamp et al., 2000; Plum et al., 2005).

**Figure 3:**
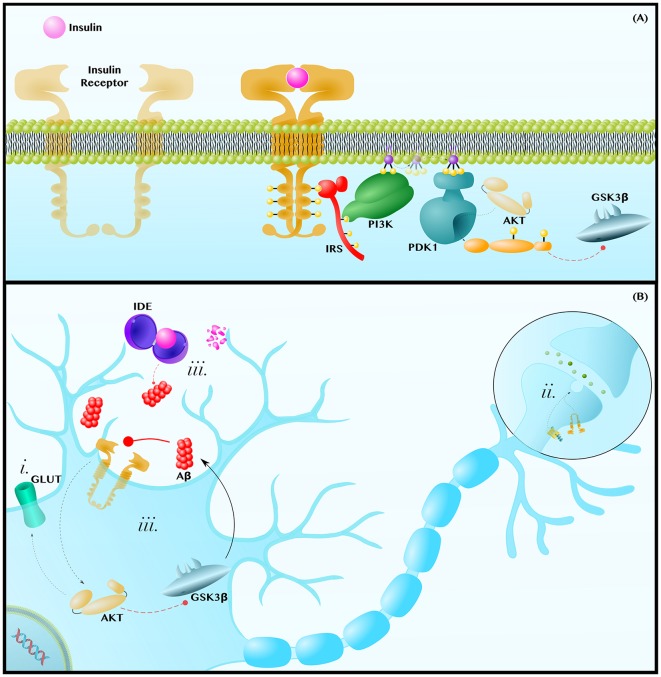
Insulin resistance exacerbates the pathology of AD. **(A)** Insulin-AKT Pathway—Insulin binds to the Insulin Receptor (IR) tyrosine kinase, which autophosphorylates and binds the adaptor protein, Insulin Receptor Substrate (IRS). IRS recruits Phosphoinositide 3-Kinase (PI3K), which phosphorylates PIP_2_ into PIP_3_. PIP_3_ diffuses along the membrane to activate Phosphoinositide-Dependent Kinase 1 (PDK1), which activates AKT. AKT phosphorylates many enzymes; this includes inhibiting GSK3β. **(B)** Insulin Resistance Contributes to Neuropathology—Insulin resistances (i) causes a decrease in AKT-mediated translocation of GLUT transporters to the membrane. This contributes to the decreased glucose metabolic rate and mitochondrial dysfunctions observed in AD and PD brains. Insulin-AKT signaling is critical in synaptic transmission, as is Wnt-signaling. Therefore, insulin resistance (ii) may synergize with dysfunctions in Wnt-signaling to decrease synaptic transmission and synapse integrity. Lastly, insulin resistance (iii) can contribute to hyperinsulinemia and the competitive inhibition of Insulin Degrading Enzyme (IDE), which also degrades Aβ. Since Aβ inhibits insulin-AKT signaling, either insulin or Aβ can establish a positive feedback loop in which Aβ inhibits insulin signaling to decrease AKT activity, increase GSK3β activity and, thus, further increase Aβ levels. Dashed and solid lines indicate regulatory mechanisms that are, respectively, impaired and enhanced in AD.

The insulin signaling cascade is initiated when insulin binds to the IR, a heterotetrameric receptor tyrosine kinase that autophosphorylates in order to recruit the adaptor protein IR Substrate (IRS). IRS subsequently recruits and activates Phosphoinositide 3-Kinase (PI3K), a lipid kinase that generates the second messenger Phosphatidylinositol (3,4,5)-trisphosphate (PIP_3_). PIP_3_ can diffuse along the membrane to activate Phosphoinositide-Dependent Kinase 1 (PDK1), which phosphorylates and activates the terminal kinase in the core of this cascade, AKT (De Meyts, 2000; Figure 3A).

AKT regulates an expansive set of pathways and processes, only some of which will be discussed in the following section. AKT (i) regulates translocation of GLUT3, the canonical neuronal glucose transporter, and of GLUT4, which is also essential in neurons (Ashrafi et al., 2017), to the plasma membrane (Grillo et al., 2009; Ferreira et al., 2011). At the axon terminal and post-synaptic density, the insulin-AKT pathway (ii) modulates catecholamine release and uptake, the trafficking of ion-gated channels, and the expression and localization of neurotransmitter receptors (Chiu et al., 2008; De Felice and Benedict, 2015). Finally, AKT (iii) is a potent GSK3β inhibitor (Zhou et al., 2014; Figure 3B). Each of these mechanisms will be discussed further in the following subsections.

### Lack of Energetic Substrates as an Exacerbating Factor for AD

Even preclinically, patients with AD show widespread impairment in glucose metabolic rates (Willette et al., 2015), a deficiency associated with decreased levels of GLUT1 and GLUT3 (Liu et al., 2008), which import glucose across the blood-brain barrier and into neurons, respectively. As the brain can only use either glucose or ketones, and ketones are not normally available as a fuel, insulin resistance and the ensuing decrease in GLUT membrane expression (Figure 3Bi) can decrease mitochondrial ATP production and all ATP-dependent maintenance processes that are critical to neuron survival (Fong et al., 2016; Blonz, 2017).

Animal models support the relevance of GLUT transporter underexpression in AD, as well as the potential involvement of dysfunctional Wnt-signaling in this process. For example, overexpression of GLUT3, which is regulated, in part, by AKT (Ferreira et al., 2011), helps rescue *Drosophila* from the morphological and behavioral features associated with Aβ toxicity (Niccoli et al., 2016). Furthermore, in a mouse model of AD, Nishida et al. (2017) demonstrated that decreased GLUT1 expression at the blood-brain barrier was associated with decreased cerebral blood flow, increased Aβ accumulation, and memory impairment. Interestingly, Wnt-signaling has been identified as necessary for GLUT1 expression at the blood-brain barrier (Daneman et al., 2009), and Pan et al. (2018) showed that inhibition of GSK3β in AD mice has precisely the opposite effects to those just described in that GSK3β inhibition increased cerebral blood flow, prevented Aβ accumulation, and rescued memory impairment. The complementary findings of the two mouse studies, in combination with the fact that Wnt ligands have been observed to increase AKT activity and neuronal glycolytic rate (Cisternas et al., 2016), hints at the possibility that dysfunctions in the insulin-AKT and Wnt-signaling pathways may cooperate to contribute to glucose metabolism deficiency in AD.

### Insulin Resistance and Wnt-Signaling in Synaptic Dysfunction

As insulin regulates the release and reception of neurotransmitters, cerebral insulin resistance can contribute to a decrease in synaptic activity and density (Abbott et al., 1999; Chiu et al., 2008; Lee et al., 2011; De Felice and Benedict, 2015; Figure 3Bii). In *Xenopus* tadpoles, the expression of a dominant-negative IR decreased excitatory post-synaptic potentials and synaptic density (Chiu et al., 2008). Conversely, activation of the insulin-AKT axis, by pharmacologically stimulating AKT or PI3K, increased synaptic density and rescued aberrant synaptic plasticity in wildtype and AD rodents (Cuesto et al., 2011; Yi et al., 2018).

At the synapse, the effects of dysfunctional Wnt-signaling have been shown to be analogous to those of dysfunctional insulin-signaling. Specifically, blocking the initiation of Wnt-signaling with DKK1 induced synaptic loss in mice (Purro et al., 2012; Marzo et al., 2016). Furthermore, as with AKT activation (Yi et al., 2018), direct pharmacological activation of Wnt-signaling was sufficient to rescue aberrant synaptic plasticity (Purro et al., 2012; Marzo et al., 2016). This, along with the suggestion of crosstalk between the Wnt and AKT pathways (Palsgaard et al., 2012; Cisternas et al., 2016), raises the possibility that insulin resistance and dysfunctional Wnt-signaling may interact to induce synaptic dysfunction in cognitive decline.

### Insulin Resistance and Aβ Can Establish a Wnt/GSK3β-Dependent Positive Feedback Loop

Insulin Degrading Enzyme (IDE) is a cytoplasmic and secreted enzyme that degrades both insulin and Aβ in the human brain (Qiu et al., 1998; Pérez et al., 2000). Accordingly, hyperinsulinemia, which is associated with an approximately two-fold increase in AD risk (Luchsinger et al., 2004), can competitively inhibit IDE-mediated Aβ degradation (Qiu et al., 1998; Pérez et al., 2000; Farris et al., 2003; Neth and Craft, 2017). In turn, Aβ can exacerbate hyperinsulinemia by inhibiting IDE and competing for IR binding (Pérez et al., 2000; Zhao et al., 2008; O’Neill, 2013).

But, even in those cases in which cerebral hyperinsulinemia does not initiate the accumulation of Aβ, a vicious cycle between Aβ and insulin-AKT signaling can arise once some degree of amyloid pathology has been established (Figure 3Biii). The De Felice group has shown that intracerebroventricular infusion of Aβ oligomers in monkeys disrupts insulin-AKT signaling in the hippocampus in a TNFα-dependent manner, leading to memory impairment (Lourenco et al., 2013). In this way, Aβ releases GSK3β from AKT-mediated inhibition and, reciprocally, GSK3β increases Aβ production via the mechanisms displayed in Figure 1B.

It is also notable that the De Felice group later showed that intracerebroventricular infusion of Aβ oligomers caused hypothalamic dysfunction and peripheral insulin resistance in mice, again in a TNFα-dependent manner. This latter finding, in conjunction with epidemiological data showing AD increases an individual’s risk of developing T2DM, suggests yet another pathological feedback loop in which systemic insulin resistance increases Aβ production, leading to Aβ-mediated hypothalamic inflammation that further exacerbates systemic insulin resistance (Clarke et al., 2015).

### The AKT Paradox

Obviously, Figure 3 is a simplification of insulin resistance pathology in the AD brain. What is not as obvious is how it is a simplification. Not only are pathways and relationships among proteins necessarily omitted, but there is also a lack of consensus on the fundamental nature of key relationships. An important and illustrative example is that AKT may be either underactive or overactive in the post-mortem human AD brain (Rickle et al., 2004; Lee et al., 2009).

While this AKT paradox remains to be resolved, one hypothesis is that the opposite dysfunctions in AKT activity are time-dependent. For example, intracellular and extracellular Aβ may have different effects on AKT activity, with intracellular Aβ (not explicitly shown in Figure 3B) accumulating well before extracellular Aβ (Magrané et al., 2005). Intracellular Aβ can interfere with the interaction between PDK1 and AKT, contributing to a decrease in AKT activity and to disease progression (Magrané et al., 2005; Lee et al., 2009). However, as extracellular Aβ builds up later, a tipping point [possibly one that is neuron-specific and heterogenous across the brain (Rickle et al., 2004)] may be reached whereby Aβ binds to IRs and constitutively overstimulates AKT (Xie et al., 2002; Zhao et al., 2008; Chiang et al., 2010). Rather than being neuroprotective, this 180° flip may be pathogenic in other ways, including saturating pathway activity, such that the pathway is no longer responsive to insulin, and inducing mTOR1-mediated IRS inhibition, thus reinforcing insulin resistance (Zhao et al., 2008; Han et al., 2018). Moreover, Aβ binding to IRs causes a dramatic migration of IRs away from neurites to the soma (Zhao et al., 2008), impairing synaptic integrity and compounding spatial complexity on top of temporal complexity.

Evidently, the AKT paradox adds a major qualification to the model presented in Figure 3B; which we presented as is for the following two reasons: (1) decreased GLUT transporter expression, decreased synaptic integrity, and increased GSK3β activity have been more consistently observed in the AD brain (Leroy et al., 2007; Liu et al., 2008; Llorens-Martín et al., 2014; Wan et al., 2014) and (2) pharmacological activators of AKT have demonstrated therapeutic efficacy in *Drosophila* and mouse models of AD (Zhang et al., 2016; Yi et al., 2018), whereas the same cannot be said for AKT inhibitors. It is important to acknowledge the AKT paradox as a representative example of the nuance present within even a single model of AD. Appreciating this nuance will help us better appreciate the true complexity of AD that arises out of an interrelationship among the models.

## An Integrated Perspective and Concluding Remarks

**Figure 4:**
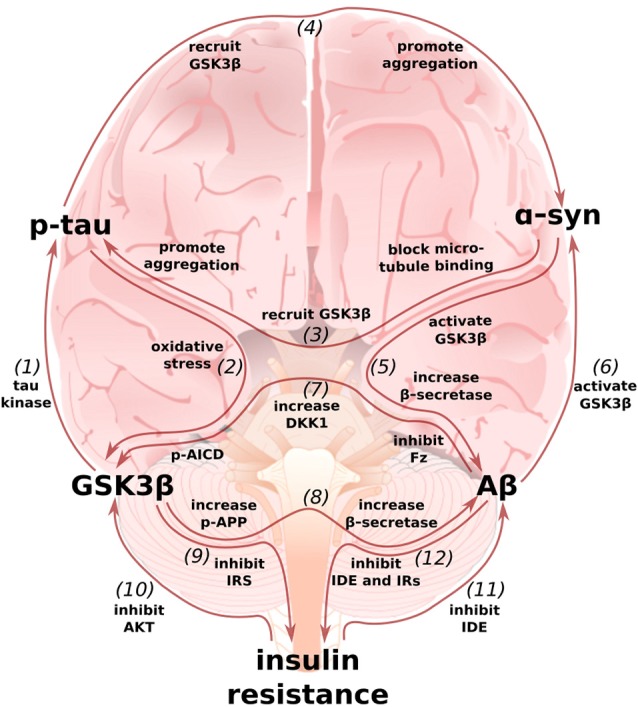
Multi-loop model of AD: an integrated perspective on the Wnt/GSK3β, α-synuclein, and type 3 diabetes hypotheses. (1) GSK3β, also known as Tau Kinase 1, phosphorylates tau (De Ferrari et al., 2014). (2) In turn, p-tau may increase GSK3β activity by inducing oxidative stress (Cente et al., 2006; Feng et al., 2013; Liu et al., 2015). (3) α-synuclein can also contribute to tau pathology by binding to tau’s microtubule binding domain (Jensen et al., 1999), recruiting GSK3β to tau, and helping to promote pathological p-tau aggregation (Gąssowska et al.’s 2014; Moussaud et al., 2014). (4) In reciprocation, tau can promote α-synuclein’s phosphorylation by GSK3β and α-synuclein aggregation (Giasson et al., 2003; Credle et al., 2015). (5) In addition to facilitating tauopathy, α-synuclein can promote Aβ production by increasing GSK3β activity (Duka et al., 2009) and β-secretase levels (Roberts et al., 2017). (6) In turn, Aβ can increase α-synuclein levels (Majd et al., 2013), possibly by stimulating GSK3β (Yuan et al., 2015). (7) Aβ can simulate GSK3β activity by inducing the expression of DKK1 and by binding to and inhibiting Frizzled (Caricasole et al., 2004; Magdesian et al., 2008). p-AICD, a by-product of Aβ production, can increase *GSK3β* gene expression (Chang et al., 2006). (8) GSK3β phosphorylates APP to enable p-AICD production and to make APP a better substrate for β-secretase (Lee et al., 2003; Acevedo et al., 2014). GSK3β overactivity and Wnt-signaling underactivity also increase β-secretase levels, further promoting the generation of Aβ (Ly et al., 2013; Parr et al., 2015). (9) GSK3β can contribute to insulin resistance by phosphorylating and inhibiting IRS1 (Lee and Kim, 2007). (10) In turn, insulin-AKT pathway dysfunction can contribute to an increase in GSK3β activity (Magrané et al., 2005; Lee et al., 2009). (11) Because insulin and Aβ are both IDE substrates, hyperinsulinemia prevents Aβ degradation (Qiu et al., 1998; Pérez et al., 2000; Farris et al., 2003; Neth and Craft, 2017; Folch et al., 2018). (12) Aβ can then further exacerbate insulin resistance by preventing insulin degradation and by binding to IRs (Pérez et al., 2000; Zhao et al., 2008; O’Neill, 2013). The above figure shows only mechanisms whereby these models feedback on one another and not those additional mechanisms whereby they cooperate to intensify AD pathology, such as may be the case for glucose transporter expression and synaptic activity (Chiu et al., 2008; Daneman et al., 2009; Ferreira et al., 2011; Purro et al., 2012; Marzo et al., 2016).

In this review article, we began by summarizing the cellular, animal, and human work that demonstrate dysfunctional Wnt-signaling can contribute to the development of AD and its two pathological hallmarks, Aβ plaques and p-tau tangles. We next described how the canonical PD-associated protein α-synuclein may be locked in pathological positive feedback loops with Aβ and tau. Finally, we discussed some of the mechanisms by which insulin resistance in the brain, “type 3 diabetes,” may contribute to development and exacerbation of AD. Throughout each section, we attempted to highlight some of the ways in which each model interacts with the others. These interrelationships, summarized in Figure 4, make it clear that the pathology of AD is not a linear cascade, nor a simple feedback loop, but rather a network of cross-talking models and overlapping vicious cycles.

Given the cooperative and reinforced nature of this complex network, it is no surprise that the prototypical monotherapeutic approach to AD has reliably failed. Certainly, drugs that target key nodes within the network, such as GSK3β inhibitors (Noble et al., 2005; Parr et al., 2012; Licht-Murava et al., 2016) or AKT activators (Zhang et al., 2016; Yi et al., 2018), have shown promise in animal models, and this important work affords us valuable mechanistic insights. However, these pre-clinical successes generally have not translated into clinical success, at least not with the same degree of efficacy. This is likely because animal models harboring distinct AD-causing mutations and dysfunctions in particular linear pathways do not accurately recapitulate the complex pathologies underlying sporadic human AD. In brief, we are proposing that the single-target silver-bullet approach to AD drug discovery is doomed to fail and that we may only be able to treat or prevent AD by developing new multifaceted treatment options.

Further complicating matters, the initial movers of sporadic human AD are likely highly individual. As examples, only about half of AD patients present with Lewy Body/α-synuclein pathology (Yan et al., 2018) and there is evidence to suggest that diabetes may specifically predispose carriers of the *ApoE4* risk allele to develop AD (Zhao et al., 2017; Folch et al., 2018). If AD is, indeed, composed of many different subtypes, then even imagining AD as a network of reinforcing positive feedback loops, as we have done here, underestimates the pathology. We may not only need multifaceted treatment options, but personalized ones.

The cost of continuing to simplify AD pathology is a continuation in the rapidly rising prevalence of AD. It is, therefore, critical that the global biomedical community take steps towards thinking more comprehensively about the mechanisms underlying AD, for only by doing so can we hope to develop multifaceted, and perhaps one day individualized, therapies to prevent or treat this devastating disease and reverse the worldwide neurodegeneration epidemic.

## Author Contributions

All authors listed have made a substantial, direct and intellectual contribution to the work, and approved it for publication.

## Conflict of Interest Statement

The authors declare that the research was conducted in the absence of any commercial or financial relationships that could be construed as a potential conflict of interest.
